# Mediating Role of Resourcefulness in the Relationship Between Illness Uncertainty and Poststroke Depression

**DOI:** 10.3389/fpsyg.2022.852739

**Published:** 2022-05-12

**Authors:** Jing Liu, Hongxia Wang, Beibei Lin, Liuqiao Ning, Danman Liu, Jufang Li

**Affiliations:** ^1^School of Nursing, Wenzhou Medical University, Wenzhou, China; ^2^First Affiliated Hospital of Wenzhou Medical University, Wenzhou, China

**Keywords:** illness uncertainty, resourcefulness, depression, mediating effect, stroke

## Abstract

**Objectives:**

To examine the association between illness uncertainty, resourcefulness, and poststroke depression (PSD) and identifies whether stroke patients’ resourcefulness plays a mediating role in the relationship between illness uncertainty and PSD.

**Methods:**

A cross-sectional study was conducted from September 2020 to April 2021. A convenience sample of 355 stroke patients was recruited. A general characteristic questionnaire, the Mishel Uncertainty in Illness Scale, the Resourcefulness Scale (RS), and the Patient Health Questionnaire-9 (PHQ-9) were used to obtain data. Descriptive analysis, Student’s *t*-test, Mann–Whitney U-test, chi-squared test, hierarchical regression analyses, Pearson correlation analysis, and mediation analysis with the PROCESS macro were used to analyze the data.

**Results:**

Illness uncertainty, resourcefulness, and PSD were significantly related to each other. Resourcefulness partially mediated the relationship between illness uncertainty and PSD.

**Conclusion:**

Illness uncertainty and resourcefulness were significantly associated with PSD, and resourcefulness played a mediating role between illness uncertainty and PSD. Interventions designed to reduce illness uncertainty and enhance resourcefulness may contribute to the prevention and improvement of PSD.

## Introduction

Poststroke depression (PSD) is the most common mental health issue found in stroke patients. According to authoritative reports, approximately 33% of stroke survivors worldwide suffer from PSD (Towfighi et al., 2017). Patients with PSD mostly present with mood swings, a lack of interest, sleep disorders, changes in appetite, etc. (Zhao et al., 2018). PSD is associated with numerous negative outcomes, such as disability, severe cognitive impairment, and poor quality of life (Kim et al., 2018; Blöchl et al., 2019; Medeiros et al., 2020). Moreover, PSD contributes to high rates of recurrence and mortality in stroke patients (Cai et al., 2019). Therefore, it is very important to identify the modifiable factors related to PSD and the mechanism by which these factors lead to PSD.

Illness uncertainty refers to an inefficient cognitive state due to an individual’s lack of ability to understand the meaning of illness-related events (Johnson Wright et al., 2009). For stroke survivors, the severe clinical manifestations and a lack of disease-related knowledge make it difficult for patients to predict the prognosis of their disease; this in turn may result in uncertainty. It has been reported that stroke patients experience moderate levels of illness uncertainty (Ni et al., 2018). Illness uncertainty, as a stressor for patients, seriously endangers their mental health (Chen et al., 2020; Verduzco-Aguirre et al., 2021). Studies have found that illness uncertainty is significantly positively correlated with depression in patients with heart failure (Chen et al., 2020) and older adults with advanced cancer (Verduzco-Aguirre et al., 2021). Although studies have shown that illness uncertainty in stroke patients may be related to PSD (Peng et al., 2016; Wei et al., 2018), the relationship between them still needs more research to be proven. In addition, there is evidence that not all individuals who experience illness uncertainty report the appearance of depressive symptoms. Some protective factors may buffer the impact of illness uncertainty on depression. Specifically, patients’ illness uncertainty not only directly promotes the occurrence of depression but also indirectly influences depression through positive psychological resources, such as hope (Cui et al., 2021) and coping with adaptive ability (Wu et al., 2020). Resourcefulness is also an important positive psychological resource for individuals, but whether it plays a mediating role in the relationship between illness uncertainty and depression has not been verified.

Resourcefulness is defined as an individual’s capability to deal with difficulties using cognitive and behavioral skills, including personal and social resourcefulness (Zauszniewski, 2016). Personal resourcefulness refers to the skill of solving problems alone, and social resourcefulness refers to the skill of solving problems by seeking help from others (Zauszniewski, 2016). With the development of positive psychology, positive psychological resources and qualities play a major role in helping individuals cope with difficult situations. Research has revealed that resourcefulness is an important psychosocial resource in protecting an individual’s wellbeing, such as in terms of adaptability (Bekhet and Zauszniewski, 2016), mental health (Zauszniewski and Burant, 2020), and quality of life (Yu et al., 2019). Previous study reported that resourcefulness is closely related to depression, and individuals with higher resourcefulness have fewer symptoms or lower levels of depression (Choi et al., 2013; Lin et al., 2017).

Moreover, resourcefulness, as a person’s ability to cope with adversity, can buffer the negative impact of stress on mental health (Zauszniewski and Burant, 2020). When an individual experiences stress, he or she may use resourcefulness, such as self-help or help-seeking strategies to respond to his or her psychological reaction. Although no research has been found to explore the relationship between illness uncertainty and resourcefulness, there is evidence that illness uncertainty may reduce individuals’ psychosocial adjustment ability (Knobf, 2011; Kazer et al., 2013), thereby aggravating depressive symptoms (Wu et al., 2020). In addition, the patient’s illness uncertainty is positively correlated with perceived stress (Moreland and Santacroce, 2018). A previous study proved that resourcefulness plays a mediating role in mitigating the impact of perceived stress on PSD (Wang et al., 2015). Therefore, we have enough reason to hypothesize that resourcefulness might function as a mediator in the relationship between illness uncertainty and PSD.

Based on the above literature review, this study aims to test the relationships between illness uncertainty, resourcefulness, and PSD, and whether resourcefulness plays a mediating role between illness uncertainty and PSD. Identifying the relationships of these variables is essential for developing corresponding interventions to improve PSD.

## Materials and Methods

### Study Design and Participants

A cross-sectional survey was conducted in a tertiary grade A hospital in southeast China from September 2020 to April 2021. Convenience sampling was used to recruit participants during their hospitalization. The participants’ inclusion criteria were as follows: (1) patients of ≥18 years of age; (2) patients diagnosed with stroke; and (3) patients who had experienced a stroke more than 7 days prior. Participant exclusion criteria were as follows: (1) patients with a history of depression or anxiety prior to stroke; (2) patients with severe aphasia; (3) patients with serious physical conditions who were unable to cooperate with the investigation; and (4) patients with impaired consciousness. A total of 370 stroke patients were invited to participate in the study, of whom 12 refused and three did not complete the questionnaire. Finally, 355 patients completed the questionnaire, and their responses were valid (effective response rate: 95.9%).

### Procedures

This study was approved by the Ethical Review Board of the data collection hospital. Face-to-face interviews were used to collect data. First, eligible participants were screened by reviewing the electronic medical records. Then, the researchers explained the purpose and content of the study to eligible participants and confirmed their willingness to participate. Before data collection, participants signed an informed consent form. After the questionnaires were distributed, participants with reading and writing skills completed the questionnaire by themselves. For participants with limited education or who were unable to fill out the questionnaire by themselves due to disease, the researchers helped them understand and fill out the questionnaire by oral translation but without any induced language. Finally, the researchers collected and checked the completeness of the questionnaires on site. If there were missing data, participants were asked to supplement their answers in time. Participants’ activities of daily living ability (ADL) scores were obtained from the electronic medical records, which were assessed by nurses on the day of data collection.

### Measurement

#### General Characteristics Questionnaire

The general characteristics questionnaire covered the participants’ sociodemographic characteristics including sex, age, spouse states, level of education, place of residence, self-evaluated economic pressure, and clinical characteristics including duration of stroke, type of stroke, location of stroke, and ADL score.

#### Mishel Uncertainty in Illness Scale

Illness uncertainty was measured by the Chinese version of the MUIS-A for adults. The scale was originally developed by Mishel (1981) and was translated into Chinese by Xu and Huang (1996). The scale contains 25 items divided into two dimensions: ambiguity (15 items) and complexity (10 items). Each item is rated on a scale of 1 (strongly disagree) to 5 (strongly agree). The total score ranges from 25 to 125, where the higher the score is, the greater the uncertainty level is. Cronbach’s alpha was measured as 0.889 for the Chinese version of the MUIS-A in a previous study (Li et al., 2020). Cronbach’s alpha of the MUIS-A was calculated as 0.860 in this study.

#### Resourcefulness Scale

The Chinese version of the resourcefulness scale (RS) was used to assess patients’ levels of resourcefulness. The scale was developed by Zauszniewski et al. (2006) and translated to Chinese by Ke et al. (2015). The scale measures 28 items with two subscales: personal (16 items) and social resourcefulness (12 items). Ratings for each item range from 0 (extremely non-descriptive of one’s behavior) to 5 (extremely descriptive of one’s behavior). The total score ranges from 0 to 140, where the higher the score is, the higher the resourcefulness level is. Cronbach’s alpha of the Chinese version of the RS was previously measured as 0.824 (Guo et al., 2019), and Cronbach’s alpha was measured as 0.771 in this study.

#### Patient Health Questionnaire-9

The Patient Health Questionnaire-9 (PHQ-9) developed by Kroenke et al. (2001) was used to assess PSD. The PHQ-9 contains nine items of two domains: cognitive/affective (four items) and somatic symptoms (five items; Johnson Wright et al., 2009). Each item is scored on a four-point scale ranging from 0 (never) to 3 (nearly every day). The total score ranges from 0 to 27, with higher scores indicating more severe depression. A total score of 5 or higher was considered to denote depression. The PHQ-9 has good internal consistency in stroke patients with a Cronbach alpha of 0.900 (Zhao et al., 2021). Cronbach’s alpha of the PHQ-9 was measured as 0.801 in this study.

### Statistical Analysis

SPSS version 23.0 was used for data analysis. Descriptive statistics were used to analyze the general characteristics of the participants and the main variables. Specifically, continuous variables were described as mean and SD or median and interquartile range (IQR), and categorical variables were presented as frequency and percentage values. Student’s *t*-test and Mann–Whitney U-test were employed to compare the differences between continuous variables, and chi-squared test was used for the comparison of categorical variables. Pearson correlation analyses were used to examine the correlations between illness uncertainty, resourcefulness, and PSD. Hierarchical regression models were used to examine the effects of illness uncertainty and resourcefulness on PSD. The general characteristics with significant differences between the PSD group and the non-PSD group were added to Model 1 to control their effect on PSD. Model 2 was built on the basis of Model 1, with illness uncertainty entered into. Model 3 was established by adding resourcefulness to Model 2. Besides, the PROCESS macro was used to analyze the mediation effect (Hayes, 2013). In this study, we take illness uncertainty as the independent variable, PSD as the dependent variable, and resourcefulness as the mediator to construct the mediation model. To control other factors that interfere with the results and improve the test’s efficiency, the general characteristics with significant differences between the PSD group and the non-PSD group were added to the covariates module. The bias-corrected 95% CI was tested with 5,000 bootstrapping resamples. If the 95% CI of the indirect effect excluded zero, the mediating role of resourcefulness was deemed significant. All significance levels were set as *p* < 0.05 (two-tailed).

## Results

### General Characteristics of the Participants

As shown in Table 1, more than half (65.60%) of the participants were male. The majority (93.24%) of the participants had spouses. Nearly 60% of the participants had a little education (primary school and below). Approximately two-thirds (66.20%) of the participants lived in rural areas. Over 60% of participants had moderate or high levels of self-evaluated economic pressure. The most common type of stroke in participants was cerebral ischemia, and nearly half of strokes were in the right hemisphere. The average age of the participants was 61.12 (SD = 10.88), the average ADL score was 73.23 (SD = 19.49), and the median (IQR) of duration of stroke was 8 (3) days.

**Table 1:** General characteristics of the sample (*N* = 355).

Variable	*n*	Percentage (%)
**Sex**		
Male	233	65.60
Female	122	34.40
**Spouse status**		
Have	331	93.24
No	24	6.76
**Level of educational**		
Primary school or below	212	59.72
Junior high school	100	28.17
High school or above	43	12.11
**Place of residence**		
Rural	235	66.20
Urban	120	33.80
**Self-evaluated economic pressure**		
Low	137	38.59
Moderate	158	44.51
High	60	16.90
**Type of stroke**		
Hemorrhagic stroke	57	16.06
Ischemic stroke	298	83.94
**Location of stroke**		
Left hemisphere	174	49.01
Right hemisphere	181	50.99

### The Differences Between PSD and Non-PSD Groups

As shown in Table 2, there were significant differences in age, level of educational, self-evaluated economic pressure, type of stroke, ADL scores, illness uncertainty, and resourcefulness among PSD group and non-PSD.

**Table 2:** The differences between non-PSD and PSD group (*N* = 355).

Variable	PSD	Non-PSD	*p*
**Sex**			
Male	57 (61.29%)	176 (67.18%)	0.305
Female	36 (38.71%)	86 (32.82%)	
Age, mean ± SD	63.30 ± 10.36	60.35 ± 10.97	0.024
**Spouse status**			
Have	85 (91.40%)	246 (93.89%)	0.410
No	8 (8.60%)	16 (6.11%)	
**Level of educational**			
Primary school or below	66 (70.97%)	146 (55.73%)	0.033
Junior high school	20 (21.51%)	80 (30.53%)	
High school or above	7 (7.53%)	36 (13.74%)	
**Place of residence**			
Rural			
Urban	61 (65.59%)	174 (66.41%)	0.886
**Self-evaluated economic pressure**			
Low	18 (19.35%)	119 (45.42%)	<0.001
Moderate	40 (43.01%)	118 (45.04%)	
High	35 (37.63%)	25 (9.54%)	
Place of residence	32 (34.41%)	88 (33.59%)	
**Type of stroke**			
Hemorrhagic stroke	25 (26.88%)	32 (12.21%)	<0.001
Ischemic stroke	68 (73.12%)	230 (87.79%)	
**Location of stroke**			
Left hemisphere	41 (44.09%)	133 (50.76%)	0.268
Right hemisphere	52 (55.91%)	129 (49.24%)	
Duration of stroke, median (IQR)	8 (3)	8 (7–9)	0.336
ADL scores, mean ± SD	56.34 ± 14.67	79.22 ± 17.37	<0.001
Illness uncertainty	78.92 ± 8.82	65.58 ± 9.96	<0.001
Resourcefulness	73.32 ± 5.99	82.38 ± 6.89	<0.001

PSD, poststroke depression; ADL, activities of daily living ability; and IQR, interquartile range.

### Correlations Among Illness Uncertainty, Resourcefulness, and Poststroke Depression

Illness uncertainty was significantly positively correlated with PSD. Illness uncertainty was significantly negatively correlated with resourcefulness. In addition, resourcefulness was significantly negatively correlated with PSD (Table 3). The mean (SD) score of illness uncertainty, resourcefulness, and PSD were 69.08 (11.31), 80.01 (7.76), 4.05 (3.29), respectively. In the 355 cases, 93 (26.20%) participants suffered from PSD as evaluated by PHQ-9.

**Table 3:** Correlations among illness uncertainty, resourcefulness, and PSD.

Variable	M ± SD	1	2	3
1 Illness uncertainty	69.08 ± 11.31	1		
2 Resourcefulness	80.01 ± 7.76	−0.542^**^	1	
3 Poststroke depression	4.05 ± 3.29	0.564^**^	−0.590^**^	1

** *p* < 0.01.

### Hierarchical Regression Analyses

Table 4 presents the hierarchical multiple regression results for PSD. Age, level of education, self-evaluated economic pressure, and ADL scores significantly affected PSD in Model 1. Self-evaluated economic pressure, ADL scores and illness uncertainty significantly affected PSD in Model 2; age, level of education no longer had an effect on PSD. The effect of illness uncertainty on PSD was reduced but still significant when resourcefulness entered into the regression in Model 3; self-evaluated economic pressure, ADL score still had a significant effect on PSD.

**Table 4:** Hierarchical regression analysis on the PSD (*N* = 355).

Variables	Model 1	Model 2	Model 3
	*β*	*β*	*β*
Age	0.030^*^	0.021	0.007
Level of education	−0.495^*^	−0.271	0.239
Self-evaluated economic pressure	1.129^**^	0.898^**^	0.627^**^
Type of stroke	−0.356	−0.479	−0.563
Score of ADL	−0.084^**^	−0.056^**^	−0.055^**^
Illness uncertainty		0.090^**^	0.040^**^
Resourcefulness			−0.175^**^
*R* ^2^	0.385	0.444	0.541
AdjustR^2^	0.376	0.435	0.532

Level of education (coded as 1 = primary school and below, 2 = junior high school, and 3 = high school and above), self-evaluated economic pressure (low = 1, moderate = 2, high = 3), type of stroke (hemorrhagic stroke = 1, ischemic stroke = 2); ADL, activities of daily living ability.

* *p* < 0.05;

** *p* < 0.01.

### Mediation of Resourcefulness in the Relationship Between Illness Uncertainty and PSD

**Figure 1:**
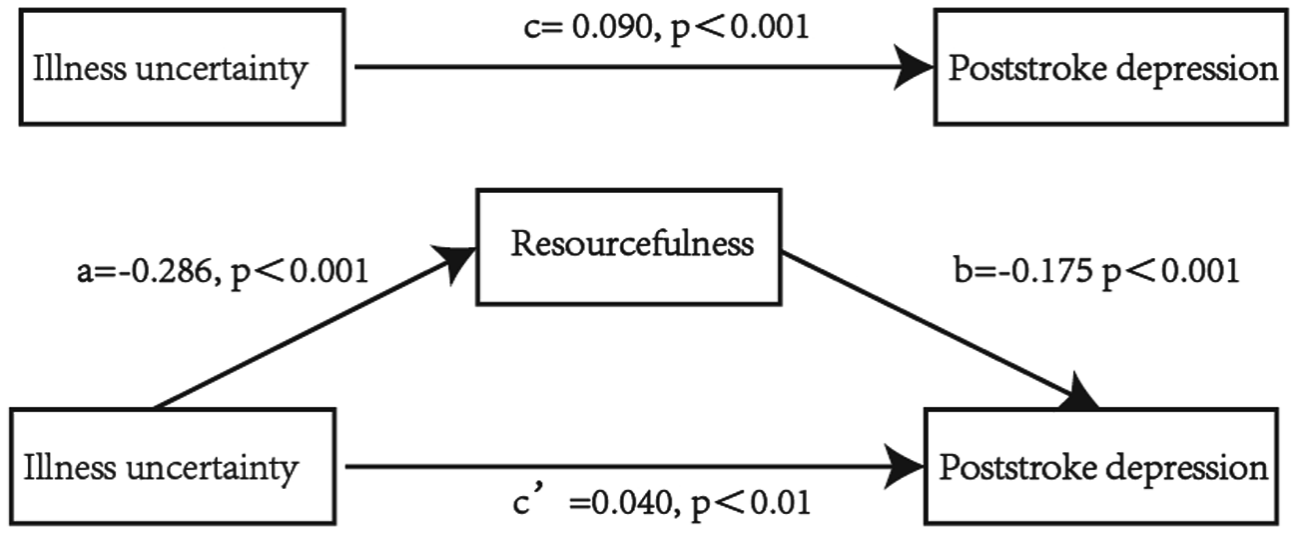
Mediating effect of resourcefulness between illness uncertainty and poststroke depression (PSD). All path coefficient was unstandardized. ^c^Path illness uncertainty—PSD (total effect). ^a*b^Path (indirect effect) and ^c’^Path (direct effect) accounted for 55.56% and 44.44% of the ^c^Path (total effect), respectively.

Table 5 shows the results of the mediation effect. After controlling for general characteristics, the total effect (path c) of illness uncertainty on PSD was measured as 0.090 (*p* < 0.001, 95% CI: 0.061, 0.118). The significant coefficients of paths a (*β* = −0.286，*p* < 0.001, 95% CI: −0.354, −0.217) and b (*β* = −0.175, *p* < 0.001, 95% CI: −0.215, −0.135) indicate that the direct effects of illness uncertainty on resourcefulness and resourcefulness on PSD were significant. In addition, the indirect effect (a * b) between illness uncertainty and PSD *via* resourcefulness was measured as 0.040 (95% CI: 0.011, 0.068). The direct effect (c’) of illness uncertainty on PSD was measured as 0.050 (*p* < 0.01, 95% CI: 0.034, 0.068), indicating that resourcefulness partially mediated the relationship between illness uncertainty in stroke patients and PSD. A simple mediating model is shown in Figure 1.

**Table 5:** Mediation of resourcefulness between illness uncertainty and PSD (*n* = 355).

Effect	Coefficient	SE	*t*	*p*	95% CI	
					LLCI	ULCI
c: Total effect of illness uncertainty on PSD	0.090	0.015	6.119	<0.001	0.061	0.118
a: Effect of illness uncertainty on resourcefulness	−0.286	0.035	−8.173	<0.001	−0.354	−0.217
b: Effect of resourcefulness on PSD after adjustment for illness uncertainty	−0.175	0.020	−8.572	<0.001	−0.215	−0.135
c’: Direct effects of illness uncertainty on PSD after adjustment for resourcefulness	0.040	0.015	2.720	0.007	0.011	0.068
a*b: Indirect effect of resourcefulness in the relationship between illness uncertainty and PSD	0.050	0.009			0.034	0.068

PSD, poststroke depression; SE, standard error; CI, confidence interval; LLCI, lower level of confidence interval; and ULCI, upper level of confidence interval.

## Discussion

This study examined the relationships between illness uncertainly, resourcefulness, and PSD in stroke patients. The results reveal that illness uncertainty, resourcefulness, and PSD were significantly related to each other. We also found that resourcefulness mediated the relationship between illness uncertainty and PSD.

### Current Status of Illness Uncertainly, Resourcefulness, and PSD

The illness uncertainly of stroke patients in this study was at an upper-middle level. This result was supported by the study of Yao (2021). The resourcefulness score of stroke patients in this study was at an intermediate level, lower than the study of Guo et al. (2019). The difference might be explained by the level of educational. In Guo et al.’s study (Guo et al., 2019), nearly 80% of the patients received junior high school education or above, but only 40% of the patients in this study had junior high school education or above. Previous research pointed out that individuals with higher education levels had higher levels of resourcefulness (Guo et al., 2019). The score of PSD in this study was 4.05 ± 3.29, which was lower than the study by Dajpratham et al. (2020). The difference might be related to the physical functional status of patients.

The physical functional status of the patients in this study was generally mildly disabled according to ADL score. However, more than half of the patients were severe disability in the Dajpratham et al.’s study (Dajpratham et al., 2020). Although the average score of PSD in this study was low, 26.6% of patients still suffered from PSD, which was similar to the results of previous study (Towfighi et al., 2017), so PSD should still be a major concern for medical staff.

### Hierarchical Multiple Regression Analyses

Multiple regression analyses showed age, level of education, self-evaluated economic pressure, and ADL score significantly predicted PSD in Model 1, which was consistent with previous findings (Guo et al., 2021). However, the effect of age and level of education on PSD was no longer significant in Model 2. The reason may be that age and level of education affected PSD through stroke patients’ illness uncertainty. Self-evaluated economic pressure, ADL score, illness uncertainty, and resourcefulness significantly predicted PSD in Model 3. Notably, self-evaluated economic pressure and ADL scores were significant influencing factors of PSD in all three models, suggesting that healthcare workers should pay more attention to stroke patients with poor economic conditions or severe physical disability. In addition, the effect of illness uncertainty on PSD was significantly decreased after adding resourcefulness, which inferred that illness uncertainty would have an indirect effect on PSD through patient resourcefulness. Furthermore, the mediation model was utilized to further verify the relationship between disease uncertainty, resourcefulness, and PSD. The results implied that illness uncertainty had a direct positive impact on PSD, and resourcefulness partially mediated the relationship between illness uncertainty and PSD.

### The Relationship Between Illness Uncertainty, Resourcefulness, and PSD in Stroke Patients

#### The Association Between Illness Uncertainty and PSD

As predicted, after controlling for general characteristics, illness uncertainty was significantly positively associated with PSD, consistent with previous studies (Peng et al., 2016; Wei et al., 2018; Chen et al., 2020). Patients with higher illness uncertainty tended to experience more severe PSD. Illness uncertainty mainly stems from a patient’s ambiguity concerning disease progression and prognosis and from the complexity of disease treatment and care-related information (Xu and Huang, 1996), which may increase a patient’s psychological stress and lead to depression (Verduzco-Aguirre et al., 2021). Furthermore, Mishel’s theory of illness uncertainty (Johnson Wright et al., 2009) notes that the impact of illness uncertainty on patients depends on how a patient evaluates illness uncertainty. When a patient regards illness uncertainty as a threat, it will lead to negative results; when it is evaluated as an opportunity, it will have a positive effect. Stroke is a shock event for stroke survivors. For stroke survivors, physical disability and risk of death make them more likely to view illness uncertainty as a threat rather than an opportunity, which may have a negative impact on patients’ psychology, such as in terms of depression.

In view of this result, assessing patients’ illness uncertainty levels and reasons for them in a timely manner and carrying out corresponding interventions may help reduce their illness uncertainty, which may ultimately improve the depressive symptoms of stroke patients. Specifically, to reduce stroke patients’ illness uncertainty, the following recommendations are made for health care professionals: on the one hand, medical staff should strengthen the health education of patients to improve patients’ illness perception; on the other hand, medical staff should communicate with patients more and answer their questions in time to improve patients’ understanding of the treatment plan, progress, and prognosis of their disease.

#### The Association Between Illness Uncertainty and Resourcefulness

After controlling for general characteristics, the illness uncertainty of stroke patients was negatively correlated with their resourcefulness in the present study. This finding is supported by a previous study showing illness uncertainty to have a negative impact on patients’ psychological adjustment ability (Li et al., 2020). For stroke patients, illness uncertainty may increase their psychological pressure, and excessive pressure will hinder them in adopting resourcefulness skills, such as positive thinking and seeking outside help. Furthermore, individuals with high level illness uncertainty generally have poor disease conditions and illness perception (Sun et al., 2021), which are internal factors of resourcefulness (Zauszniewski, 2016). Thus, higher illness uncertainty leads to a lower level of resourcefulness. This reminds health professionals that reducing the illness uncertainty of stroke patients may be an important means to improve their resourcefulness.

#### The Association Between Resourcefulness and PSD

After controlling for general characteristics and levels of illness uncertainty, resourcefulness was negatively correlated with PSD among stroke patients. This result is aligned with previous studies that show resourcefulness to be a protective factor for depression (Wang et al., 2015; Lin et al., 2017). Resourcefulness is divided into personal and social resourcefulness, both of which are protective factors for individual mental health (Zauszniewski, 2016). Specifically, individuals high in personal resourcefulness are good at using internal resources, such as self-control, positive self-talk, and self-evaluation, to effectively cope with stressful events. Individuals high in social resourcefulness have a strong ability to seek help from external resources, that is, social support. Social support not only provides material assistance to patients but also enables patients to have positive experiences, such as care and respect, preventing negative emotions, including depression (Volz et al., 2016). Therefore, developing resourcefulness skills may be an effective means to manage PSD. Previous studies have confirmed that resourcefulness training as a cognitive-behavioral intervention is an effective means to promote the development of resourcefulness skills (Toly et al., 2014; Bekhet and Zauszniewski, 2016). In the future, medical staff can consider carrying out resourcefulness training to prevent and manage PSD.

#### Resourcefulness Mediated the Relationship Between Illness Uncertainty and PSD

Notably, resourcefulness played a partially mediating role in the relationship between illness uncertainty and PSD, which indicates that high illness uncertainty among stroke patients may worsen PSD. However, this association may be reversed by enhancing resourcefulness. Previous studies have shown that the higher a patient’s illness uncertainty is, the greater the perceived stress level becomes (Moreland and Santacroce, 2018); the higher a patient’s resourcefulness is, the stronger his or her ability to adapt becomes (Bekhet and Zauszniewski, 2016). Although no prior studies have examined the mediating effect of resourcefulness on the relationship between illness uncertainty and PSD, the findings of this study are similar to the results of previous studies that show the mediating effects of resourcefulness on the relationship between perceived stress and depression in stroke patients (Wang et al., 2015) and the mediating effects of adaptive ability on the relationship between illness uncertainty and psychological stress response among patients in emergency observation room settings (Wu et al., 2020). This finding is also in line with the view of resourcefulness theory that resourcefulness mediates the impact of situational factors on outcomes (Zauszniewski, 2016). In addition, this finding is supported by Lazarus’s coping model, which indicates that when faced with stressors (such as illness uncertainty), individuals with strong coping capabilities (such as resourcefulness) can effectively control the development of an event, thereby maintaining a good mental state (Lazarus, 1992). This finding is valuable in that it strengthens our understanding of the path of PSD generation by examining the relationship between patient uncertainty and resourcefulness. In addition, this finding further demonstrates that improving patients’ resourcefulness is an important means to help patients cope with psychological problems.

### Limitations and Avenues for Future Research

Several limitations of this study should be noted. First, the study participants were recruited using convenience sampling, which may lead to significant selective bias. A randomized sampling study should be conducted in the future. Second, our participants were recruited from one hospital, which might limit the generalizability of the findings. A multicenter study is planned to increase the generalizability of the study results. Finally, other variables, such as social support and self-efficacy, that may be related to PSD were not included in this study. Further research is under the way to include more variables to gain a more comprehensive understanding of the pathogenesis of PSD.

## Conclusion

This study demonstrated that illness uncertainty and resourcefulness were significant influencing factors of PSD, and resourcefulness mediates the relationship between illness uncertainty and PSD. Interventions designed to reduce illness uncertainty and enhance resourcefulness may contribute to the prevention and improvement of PSD.

## Data Availability Statement

The raw data supporting the conclusions of this article will be made available by the authors, without undue reservation.

## Ethics Statement

The studies involving human participants were reviewed and approved by Ethics Committee of the First Affiliated Hospital of Wenzhou Medical University. The patients/participants provided their written informed consent to participate in this study.

## Author Contributions

JiL: conceptualization, methodology, and manuscript preparation and revision. JuL: conceptualization, methodology, and manuscript revision. HW: acquisition of data and revising it critically for important intellectual content. BL: acquisition of data and writing—review and editing. LN and DL: acquisition of data and writing—review. All authors contributed to the article and approved the submitted version.

## Funding

This study was supported by the National Natural Science Foundation of China (grant number: 71804134), the Natural Science Foundation of Zhejiang Province (grant number: LQ18G030006), the Project of Humanities and Social Sciences from the Ministry of Education in China (grant number: 18YJCZH078), and the Health Commission of Zhejiang Province (grant number: 2021KY780).

## Conflict of Interest

The authors declare that the research was conducted in the absence of any commercial or financial relationships that could be construed as a potential conflict of interest.

## Publisher’s Note

All claims expressed in this article are solely those of the authors and do not necessarily represent those of their affiliated organizations, or those of the publisher, the editors and the reviewers. Any product that may be evaluated in this article, or claim that may be made by its manufacturer, is not guaranteed or endorsed by the publisher.
